# Dirac solitons in optical microresonators

**DOI:** 10.1038/s41377-020-00438-w

**Published:** 2020-12-23

**Authors:** Heming Wang, Yu-Kun Lu, Lue Wu, Dong Yoon Oh, Boqiang Shen, Seung Hoon Lee, Kerry Vahala

**Affiliations:** 1 T. J. Watson Laboratory of Applied Physics, California Institute of Technology, Pasadena, CA 91125 USA; 2 Present Address: Research Laboratory of Electronics, MIT-Harvard Center for Ultracold Atoms, Department of Physics, Massachusetts Institute of Technology, Cambridge, MA USA; 3 Present Address: Rockley Photonics Inc., Pasadena, CA USA; 4 Present Address: Apple Inc., Cupertino, CA USA

**Keywords:** Solitons, Photonic devices

## Abstract

Mode-coupling-induced dispersion has been used to engineer microresonators for soliton generation at the edge of the visible band. Here, we show that the optical soliton formed in this way is analogous to optical Bragg solitons and, more generally, to the Dirac soliton in quantum field theory. This optical Dirac soliton is studied theoretically, and a closed-form solution is derived in the corresponding conservative system. Both analytical and numerical solutions show unusual properties, such as polarization twisting and asymmetrical optical spectra. The closed-form solution is also used to study the repetition rate shift in the soliton. An observation of the asymmetrical spectrum is analysed using theory. The properties of Dirac optical solitons in microresonators are important at a fundamental level and provide a road map for soliton microcomb generation in the visible band.

## Dirac solitons: Equation describes new properties

The intriguing properties of a new type of ultrashort light pulse in microresonators are described. Called a Dirac soliton, Heming Wang and colleagues at the California Institute of Technology say that understanding the properties of Dirac solitons is important at the fundamental level and also for providing a road map for generating ultrashort soliton pulses in the visible light range. The latter is relevant for applications such as miniature optical clocks and medical imaging techniques, for example. The solitons are generated in micron-scale devices called microresonators. And the soliton properties are controlled by the microresonator shape and choice of material. Wang and his colleagues also called attention to the importance of broadband nonlinear coupling in the microresonators for generation of the new Dirac soliton, which their equations show exhibits polarization twisting and asymmetrical optical spectra.

## Introduction

Soliton mode locking in microresonators^1^ provides a pathway for the miniaturization of frequency comb systems^2^. The dissipative solitons^3^ formed in the resulting microcombs^4^ are coherently pumped^5^ and were first observed in optical fibre cavities^6^. In microresonators, such Kerr solitons (KSs) have been realized in a wide range of geometries and material systems^7–14^. Soliton microcomb devices have been tested in diverse system demonstrations, including spectroscopy^15–17^, coherent communications^18^, range detection^19–21^, optical frequency synthesis^22^, exoplanet studies^23,24^, and optical clocks^25^. Progress towards integration of the microcomb with pump and other control functions is also being made^26–28^. Modal coupling, wherein distinct mode families experience frequency degeneracy analogous to an energy level crossing^29^, is an important feature of soliton formation in microresonators. Such crossings impart structure to the soliton spectral envelope^30^ and are responsible for an intriguing range of microcomb phenomena of both scientific and technical importance, including dispersive wave emissions^9,31^, dark soliton formation^32^, pump noise isolation^33^, improved pumping efficiency^34,35^, and dispersion engineering for near-visible emissions^36,37^.

Here, a new type of soliton in microresonators, termed Dirac solitons (DSs), is shown to result from broadband modal coupling. The name originates from the nonlinear Dirac equations, which govern the dynamics of these solitons and are discussed below. A similar soliton has been theoretically studied in fibre Bragg gratings^38,39^ and later experimentally observed^40^. In these Bragg solitons, forward and backward propagating waves are coupled by a periodic structure, and a Dirac-like model has been applied to understand these systems^41,42^. As shown recently for broadband coupling in a dimer resonator system^43^, the co-existence of coupling and nonlinearity changes the solution behaviour qualitatively, and a full understanding requires a non-perturbative approach. We show that broadband nonlinear coupling results in a range of new phenomena in the Dirac soliton system, including polarization twisting along the soliton and asymmetrical soliton comb spectra. A closed-form expression for DSs is derived by solving the Lugiato-Lefever equation (LLE)^5,44^ augmented with mode coupling. Curiously, the requisite coupling conditions for DS generation have been obtained experimentally for near-visible^36^ and 1-μm-band^37^ soliton generation. As shown here, these experiments were performed in a regime where Dirac solitons collapse into conventional Kerr solitons. New data from the 778-nm-band measurement featuring asymmetrical spectra will be presented, showing initial deviation away from conventional Kerr soliton behaviour.

## Results

### Polarization mode coupling and coupled LLEs

**Fig. 1:**
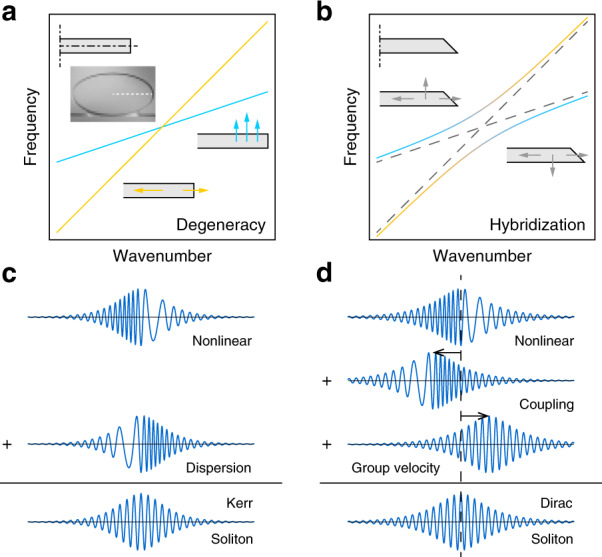
Principle of mode hybridization and Dirac solitons. **a** For a symmetric resonator cross section (top-left insets), TE and TM modes within the resonator can become accidentally degenerate at the same wavenumber. The bottom (right) inset depicts the TE (TM) mode electric field directions. **b** For an asymmetric resonator cross section (top-left inset), degeneracy is lifted, and an avoided crossing is created. The left and right insets depict the hybridized mode electric field directions. **c** Schematic of balancing nonlinear and dispersion effects for a KS. **d** Schematic of balancing nonlinear, coupling and group velocity difference effects for one of the components in a DS

To illustrate the features of DSs, we consider the specific case of a circularly symmetric (whispering-gallery) resonator that has an initial reflection plane of symmetry in the plane of the resonator. The resulting geometry supports transverse-electric (TE) and transverse-magnetic (TM) mode families (Fig. 1a) that are symmetrical and anti-symmetrical, respectively, with respect to the reflection plane. A pair of TE and TM modes can have accidental degeneracy for a particular wavenumber. By breaking the reflection geometry, it becomes possible to lift the degeneracy and create an avoided crossing^36^. In effect, the original modes are strongly coupled, and the eigenmodes of the non-symmetric system are hybrid modes, as shown in Fig. 1b. Loosely speaking, the resulting hyperbolic shape of the eigenfrequency dispersion creates an anomalous dispersion window that is suitable for soliton generation. However, we note that this dispersion is not associated with a single mode family across the entire avoided crossing. Indeed, the mode composition changes when the wavenumber increases, evolving from TM to TE mode for the upper branch or vice versa for the lower branch.

The standard form of the LLE for one transverse mode family (denoted by mode 1) describes the temporal soliton dynamics in a microresonator^44^:

1 $$\frac{{\partial E_1}}{{\partial t}} = ( - i\delta \omega - \frac{{\kappa _1}}{2})E_1 - i\hat L_1E_1 + ig_{11}|E_1|^2E_1 + f_1$$

Here, *E*_1_ is the slowly varying amplitude in a co-moving frame normalized to optical energy, defined via **E**_1_ = *E*_1_**A**_1_, where **E**_1_ is the electric field and **A**_1_ is the normalized vector field distribution. The frequency detuning *δω* = *ω*_c_ − *ω*_p_ is the frequency difference between the cavity resonant frequency *ω*_c_ and pump frequency *ω*_p_. The Kerr nonlinear coefficient is $g_{11} = n_{(2)}\omega _{\mathrm{c}}c{\mathrm{/}}(n^2V_{11})$, with speed of light in vacuum *c*, refractive index *n*, Kerr nonlinear index *n*_(2)_ and mode volume $V_{11} = ({\int} {|{\mathbf{A}}_1|^2dV} )^2{\mathrm{/}}{\int} {|{\mathbf{A}}_1|^4dV}$. *κ*_1_ is the energy loss rate, and *f*_1_ is the pumping term for mode 1. The linear dispersion operator $\tilde L_1$ describes mode dispersion and can be Taylor expanded as $\hat L_1 \approx - iD_{1,1}\partial _\theta - D_{2,1}\partial _\theta ^2{\mathrm{/}}2$, where *θ* is the angular coordinate and *D*_1,1_/(2*π*) and *D*_2,1_/(2*π*) are the free spectral range (FSR) and second-order dispersion (proportional to the group velocity dispersion (GVD)), respectively, for mode 1. In the case of a conservative system (*κ*_1_ = 0 and *f*_1_ = 0) and *D*_1,1_ = 0 (i.e., choosing a co-moving reference frame), the exact soliton solution to Eq. (1) is given by:

2 $$E_1 = \sqrt {\frac{{2\delta \omega }}{{g_{11}}}} {\mathrm{sech}}\left( {\sqrt {\frac{{2\delta \omega }}{{D_{2,1}}}} \theta } \right)$$

which is also commonly used as an ansatz to describe a KS^7^.

To generalize the above LLE to the coupled pair of TE and TM modes (modes 1 and 2, respectively), we introduce mode coupling as well as cross-phase modulation into the equations. The following two-mode coupled LLE results:

3 $$\begin{array}{l}\frac{{\partial E_1}}{{\partial t}} = ( - i\delta \omega - \frac{{\kappa _1}}{2})E_1 + ig_{\mathrm{c}}E_2 - \delta D_1\frac{{\partial E_1}}{{\partial \theta }}\\ + i(g_{11}|E_1|^2E_1 + g_{12}|E_2|^2E_1) + f_1\\ \frac{{\partial E_2}}{{\partial t}} = ( - i\delta \omega - \frac{{\kappa _2}}{2})E_2 + ig_{\mathrm{c}}E_1 + \delta D_1\frac{{\partial E_2}}{{\partial \theta }}\\ + i(g_{22}|E_2|^2E_2 + g_{12}|E_1|^2E_2) + f_2\end{array}$$

Here, *g*_c_ > 0 gives the coupling rate between the two (originally uncoupled) modes, $g_{ij} = n_{(2)}\omega _{\mathrm{c}}c{\mathrm{/}}(n^2V_{ij})$ (*i*, *j* = 1, 2), and the mode volumes *V*_11_ and *V*_22_ and cross mode volume *V*_12_ are defined as $V_{ij} = ({\int} {|{\mathbf{A}}_i|^2dV} {\int} {|{\mathbf{A}}_j|^2dV} ){\mathrm{/}}{\int} {|{\mathbf{A}}_i|^2|{\mathbf{A}}_j|^2dV}$. A reference frequency (relative to which all frequencies are measured) is chosen as the degeneracy frequency. In the symmetric co-moving frame (moving at a speed corresponding to $\bar D_1 = (D_{1,1} + D_{1,2}){\mathrm{/}}2$), the group velocities of the two resulting hybrid modes become anti-symmetric with $\delta D_1 = |D_{1,1} - D_{1,2}|/2$. Here, second- and higher-order dispersions are ignored, as the coupling-induced dispersion of the eigenfrequency is typically one order of magnitude larger than the intrinsic mode dispersion. The nonlinear terms include self-phase and cross-phase modulation. Other four-wave mixing terms that induce nonlinear coupling, such as $|E_1|^2E_1E_2^ \ast$ and $E_1^2(E_2^ \ast )^2$, have been dropped because these are either forbidden by reflection symmetry or strongly suppressed by the phase mismatching of the underlying modes.

The LLE Eq. (1) (without loss and pump terms) is known as the nonlinear Schrödinger equation in theoretical physics. Similarly, the coupled LLE Eq. (3) presented here are a generalization of the nonlinear Dirac equations. When only cross-phase modulation is considered (*g*_11_ = *g*_22_ = 0), Eq. (3) reduces to the massive Thirring model in quantum field theory^45^, which is known to support Dirac solitons^46,47^. With equal but nonzero self-phase modulations (*g*_11_ = *g*_22_), the Bragg soliton solution^38,39^ is recovered, which has been realized in fibre Bragg grating systems^40^. On the other hand, when second-order dispersion is present and is much stronger than the effect induced by linear inter-mode coupling within the band being considered, Eq. (3) becomes the vector soliton model in birefringent systems, where soliton solutions are also known^48–50^. We note that the vector soliton relies on both modes having anomalous dispersion, while anomalous dispersion is not required in the DS model.

Before proceeding to solve Eq. (3), it is helpful to understand why a DS solution exists in the absence of second-order dispersion. The conventional KS is a delicate balance of the Kerr nonlinear effect, which creates chirping within a pulse, and the anomalous mode dispersion, which cancels the chirping effect (Fig. 1c). In the DS system, the hyperbolic-shaped upper-branch eigenfrequency spectrum (as in Fig. 1b) resembles a spectrum with anomalous dispersion. While this “dispersion” plays the same role as conventional dispersion and constitutes the foundation for the generation of the DS, this viewpoint only holds when the pumping frequency (and soliton spectrum) is close to this branch. Indeed, in this case, it will be shown that it reduces to the conventional KS. In general, and as noted in the introduction, dispersion is only locally well-defined in this spectrum, because the mode composition of the hybrid mode can change rapidly with respect to the wavenumber. Correspondingly, the dispersion interpretation fails for the general DS, and the coupling effect must be treated non-perturbatively. These rapid composition changes in the hybridized modes redistribute pulse energy in the frequency domain and produce a new contribution to chirping within the pulse, which manifests as phase differences between the two-mode components of the pulse. Coupling then makes the two components interfere differently at different positions and leads to both chirping and pulse shifting. These effects are delicately cancelled by nonlinear effects and group velocity differences, respectively (Fig. 1d), and maintain the DS pulse shape without anomalous dispersion. In the language of field theory, anomalous mode dispersion provides a positive “mass” for the KS field, which then becomes a well-defined non-relativistic field theory. The “mass” of the DS field is the inter-mode coupling, and the mode spectrum corresponds to a relativistic field theory.

### Closed-form soliton solutions

To obtain an analytical solution for the coupled LLE, we consider a conservative system by setting *κ*_1_ = *κ*_2_ = 0 and *f*_1_ = *f*_2_ = 0. Equation (3) can then be solved by finding the invariants associated with the system (see ‘Materials and methods’). The closed-form single bright soliton solution for Eq. (3), without periodic boundary conditions, can be obtained as:

4 $$\begin{array}{c}E_{1,2} = \pm \sqrt {\frac{{2g_{\mathrm{c}}}}{G}} \sqrt {1 - \tilde \xi ^2} (\frac{{\delta D_1 \pm v}}{{\delta D_1 \mp v}})^{1/4}\\ \frac{{\left[ {{\mathrm{cosh}}\left( {2\sqrt {1 - \tilde \xi ^2} \tilde \theta } \right) - \tilde \xi } \right]^{ \pm \gamma /2}}}{{\left[ {\sqrt {1 - \tilde \xi } {\mathrm{cosh}}\left( {\sqrt {1 - \tilde \xi ^2} \tilde \theta } \right) \mp i\sqrt {1 + \tilde \xi } {\mathrm{sinh}}\left( {\sqrt {1 - \tilde \xi ^2} \tilde \theta } \right)} \right]^{1 \pm \gamma }}}\\ {\mathrm{exp}}\left( { - i\frac{v}{{\delta D_1}}\tilde \xi \tilde \theta } \right)\end{array}$$

$$\begin{array}{l}G = \frac{{\delta D_1 + v}}{{\delta D_1 - v}}\frac{{g_{11}}}{2} + \frac{{\delta D_1 - v}}{{\delta D_1 + v}}\frac{{g_{22}}}{2} + g_{12},\gamma = \frac{1}{G}\left( {\frac{{\delta D_1 + v}}{{\delta D_1 - v}}g_{11} - \frac{{\delta D_1 - v}}{{\delta D_1 + v}}g_{22}} \right)\\ \tilde \xi = \frac{{\delta D_1}}{{\sqrt {\delta D_1^2 - v^2} }}\frac{{\delta \omega }}{{g_c}},\tilde \theta = \frac{{g_c}}{{\sqrt {\delta D_1^2 - v^2} }}\theta \end{array}$$

where *E*_1_ (*E*_2_) takes the upper (lower) sign in all instances of ± or ∓, *v* is the repetition rate shift in the symmetric co-moving frame, *G* is the combined nonlinear coefficient, *γ* is a phase exponent related to *v*, $\tilde \xi$ is the reduced detuning and $\tilde \theta$ is the reduced coordinate. While dark soliton solutions and bright soliton on a background solutions can also be found in the same conservative system (see Supplementary Information), we will focus on this bright soliton solution and refer to it as the DS. In the following discussion of DS properties, we take the special case *g*_11_ = *g*_22_ (i.e., additional exchange symmetry between the modes) and *v* = 0 (i.e., the pulse is stationary in the symmetric co-moving frame), and the general solution simplifies to:

5 $$E_{1,2} = \sqrt {\frac{{2(g_{\mathrm{c}}^2 - \delta \omega ^2)}}{{(g_{11} + g_{12})g_{\mathrm{c}}}}} \frac{1}{{ \pm \sqrt {g_{\mathrm{c}} - \delta \omega } {\mathrm{cosh}}\left( {\sqrt {g_{\mathrm{c}}^2 - \delta \omega ^2} \theta {\mathrm{/}}\delta D_1} \right) - i\sqrt {g_{\mathrm{c}} + \delta \omega } {\mathrm{sinh}}\left( {\sqrt {g_{\mathrm{c}}^2 - \delta \omega ^2} \theta {\mathrm{/}}\delta D_1} \right)}}$$

**Fig. 2:**
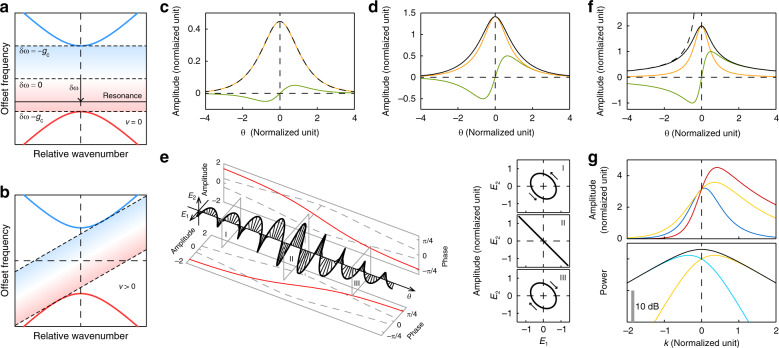
Closed-form solution of Dirac solitons in microresonators. **a** Resonance diagram showing two branches of hybrid modes, the allowed range for the soliton resonance line when *v* = 0 (shaded area), and the three special cases of detuning discussed in the text. **b** Same as (**a**) but shows only the range for the soliton resonance line with an arbitrary fixed positive repetition rate shift, *v*. **c** Real part (orange line) and imaginary part (green line) of the *E*_1_ component of the DS at *δω* = −0.9*g*_c_. The normalization scheme used for the plot is *g*_c_ = 1, *δD*_1_ = 1 and *g*_11_ + *g*_12_ = 1. The black dashed curve shows the corresponding KS profile for comparison. **d** Real part (orange line), imaginary part (green line) and norm (black line) of the *E*_1_ component of the DS at *δω* = 0. The normalization scheme used for the plot is the same as that in (**c**). **e** Polarization twist of DS at *δω* = 0. The two projections show the slowly varying amplitude envelope (grey dashed lines, left scale) and phase relative to the pulse centre (red solid lines, right scale) of the field components. The right insets show the polarization states at the three different spatial slices marked on the plot. The arrow indicates the direction of state change over time. **f** Real part (orange line), imaginary part (green line) and norm (black solid line) of the *E*_1_ component of the DS at *δω* = *g*_c_. The normalization scheme used for the plot is the same as that in (**c**). The black dashed line shows the 1/*θ* asymptote for the envelope. **g** Upper panel: frequency domain amplitudes for the *E*_1_ component of the DS at *δω* = −0.8*g*_c_ (blue line), *δω* = 0 (yellow line), and *δω* = 0.8*g*_c_ (red line), plotted on a linear scale. Lower panel: frequency domain power distribution for the *E*_1_ component (yellow line), *E*_2_ component (cyan line) and combined (black line) DS at *δω* = 0, plotted on a log scale. The normalization scheme used for the plot is the same as that in (**c**)

Figure 2a shows the offset frequency for the hybrid modes, defined as $\omega _{{\mathrm{off}}} = \omega _k - \omega _{\mathrm{c}} - k\bar D_1$ where *k* is the relative wavenumber (the difference between the absolute wavenumber and the wavenumber at the degeneracy point) and *ω*_*k*_ is the mode eigenfrequency at *k*. Due to the square roots in the special solution (Eq. (5)), the soliton detuning range can lie only in the band gap created by the avoided crossing. This phenomenon can be intuitively understood, as none of the comb lines can have the same frequency as the resonator modes, which would otherwise create infinite amplitudes on the modes due to perfect resonance with no loss. Geometrically, the resonance of the soliton can be described by the linear equation *ω*_DS_ = −*δω* + *vk*, and the line cannot intersect the two hyperbolas of mode frequencies on the mode spectrum plot. The same also holds for the general solution (Eq. (4)) and is depicted in Fig. 2b (see ‘Materials and methods’). As a result, the soliton cannot have a group velocity faster than the first mode or slower than the second mode. This argument does not apply to dark solitons and bright solitons on a background, where the resonance line always intersects the mode spectrum (see Supplementary Information).

To understand the properties of the DS, we consider some special cases of detuning (marked in Fig. 2a). The first case is when *δω* approaches −*g*_c_, where the resonance line is close to the upper branch. By taking appropriate limits, Eq. (5) reduces to:

6 $$E_{1,2} \approx \pm \sqrt {\frac{{2(g_{\mathrm{c}} + \delta \omega )}}{{g_{11} + g_{12}}}} {\mathrm{sech}}\left( {\sqrt {\frac{{2g_{\mathrm{c}}(g_{\mathrm{c}} + \delta \omega )}}{{\delta D_1^2}}} \theta } \right)$$

which is exactly in the form of a conventional KS. A comparison of the exact DS near *δω* = −*g*_c_ and the limiting KS is shown in Fig. 2c. The appearance of the sech-shaped KS here is not a coincidence. The effective nonlinear coefficient for a single component is *g*_11_ + *g*_12_ (using $|E_1|^2 = |E_2|^2$), the effective detuning is *g*_c_ + *δω*, and the curvature of the eigenfrequency (the hybrid-mode equivalent of the second-order dispersion) is $\delta D_1^2{\mathrm{/}}g_{\mathrm{c}}$, as derived from coupled mode theory. The reduction of a DS to a KS is straightforward when these quantities are substituted into the KS solution. Thus, if the DS is close to the resonance (which is usually the case when the hybridization coupling *g*_c_ is large), the eigenfrequency spectrum is locally equivalent to a single mode in terms of dispersion, and mode composition differences for different wavenumbers can be ignored. This phenomenon also applies to the general solution (Eq. (4)) by explicitly reducing the coupled LLE to a single-mode LLE, and the truncation errors can also be estimated (see ‘Materials and methods’).

The second case is when *δω* = 0, where the resonance line passes through the degeneracy point. In this case, Eq. (5) simplifies to:

7 $$E_{1,2} = \sqrt {\frac{{2g_{\mathrm{c}}}}{{g_{11} + g_{12}}}} \frac{1}{{ \pm {\mathrm{cosh}}\left( {g_{\mathrm{c}}\theta {\mathrm{/}}\delta D_1} \right) - i{\mathrm{sinh}}\left( {g_{\mathrm{c}}\theta {\mathrm{/}}\delta D_1} \right)}}$$

and begins to deviate from a hyperbolic secant shape (Fig. 2d). With this analytical solution, it becomes apparent that each component of the wave packet has an overall phase shift when *θ* goes from −∞ to ∞ (*π*/2 in this special case). This phase twist within the pulse contributes to the chirping and shifting of the soliton pulse when they are coupled together, as discussed in the previous section. If pulse polarization is considered, it also twists from the start of the pulse to the end of the pulse (Fig. 2e).

The last case we consider is when *δω* approaches *g*_c_, where the resonance line moves towards the lower branch and maximum red detuning is approached. As this phenomenon occurs, the exponential tails of the soliton decay at an increasingly slower rate until finally in the limit *δω* → *g*_c_ Eq. (5) becomes:

8 $$E_{1,2} \approx \sqrt {\frac{{g_{\mathrm{c}}}}{{g_{11} + g_{12}}}} \frac{2}{{ \pm 1 - 2ig_{\mathrm{c}}\theta {\mathrm{/}}\delta D_1}}$$

showing that the solution decays polynomially rather than exponentially when *θ* → ∞ (Fig. 2f). The resulting polynomial tails can potentially enable long-range interactions of the DS.

We now turn to the frequency domain profile for Eq. (5) by the Fourier transform, which can also be expressed in closed form using contour integration:

9 $${\int\nolimits_{ - \infty }^\infty} {E_{1,2}} e^{ - ik\theta }d\theta = \pm \pi \sqrt {\frac{{\delta D_1^2}}{{(g_{11} + g_{12})g_c}}} {\mathrm{sech}}\,\tilde k\exp \left[ { \pm \frac{{{\mathrm{arccos}}( - \delta \omega {\mathrm{/}}g_c)}}{\pi }\tilde k} \right]$$

where *k* is the relative wavenumber and $\tilde k = \pi \delta D_1k{\mathrm{/}}(2\sqrt {g_{\mathrm{c}}^2 - \delta \omega ^2} )$. Apart from the usual sech-shaped envelope, the extra exponential factor causes the spectrum of each component to become asymmetrical around *k* = 0 (Fig. 2g). This phenomenon can be explained by different mode compositions on the different sides of the spectrum. We note that the spectrum for the total power is still symmetric, which is consistent with the soliton carrying no total momentum in the symmetric co-moving frame. In the general *v* ≠ 0 case, the spectrum for the total power is expected to become asymmetric around the centre frequency of the soliton.

### DS with dissipation and repetition rate shifts

In addition to being an exact solution to the conservative system, the solution (Eq. (4)) also serves as a DS ansatz for dissipative cases, similar to how a KS can be approximated by a sech-shaped soliton on a background^7^. As an example, we study the repetition rate shifts associated with the DS when externally pumped by continuous waves. Accordingly, we do not require *g*_11_ = *g*_22_ or *v* = 0 and return to work with the general solution (Eq. (4)).

The repetition rates of ideal KSs remain constant when pumped in the same mode while the detuning changes, as formulated by the standard LLE. In contrast, real-world KSs may experience additional nonlinear effects, including dispersive wave backactions^33^ or Raman effects^51^, which lead to centre frequency changes and repetition rate shifts. The mode hybridization process is similar to a mode crossing in dispersive wave generation where two modes are strongly coupled, and therefore, the DS is also expected to experience repetition rate shifts. As the repetition rate shift parameter *v* is free in the conservative case, we need to find the conditions that determine *v* when dissipation is present.

By calculating the momentum integral of the solution, the following criterion is obtained (see ‘Materials and methods’):

10 $${\int} {\left( {\kappa _1|E_1|^2\frac{{\partial {\mathrm{arg}}E_1}}{{\partial \theta }} + \kappa _2|E_2|^2\frac{{\partial {\mathrm{arg}}E_2}}{{\partial \theta }}} \right)} d\theta = 0$$

where arg is the argument function and *E*_1,2_ should be substituted by the DS solution. According to the above criterion, the phase twist of each component is essential in determining the repetition rate shift. Intuitively, this concept can be understood as follows: the pulse cannot carry any net momentum in the reference frame of the pumping, and any additional momentum will be damped out by the dissipation. All the above integrations can be carried out in closed form, leading to an equation in *v*, which can then be solved as the repetition rate shift.

**Fig. 3:**
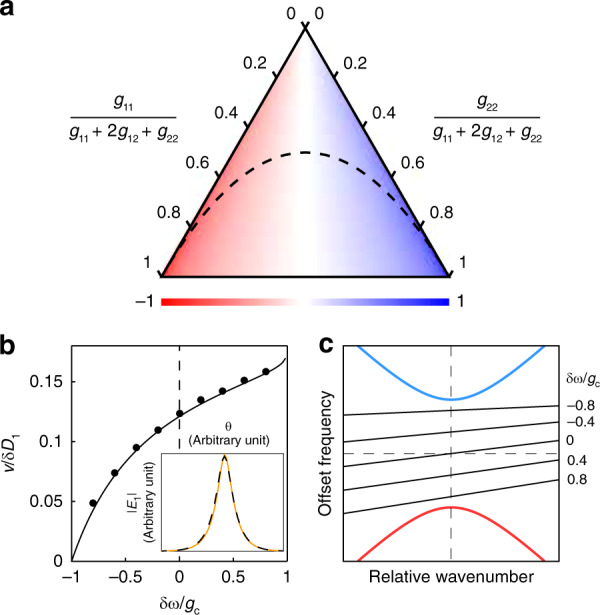
Repetition rate shifts in the DS. **a** Ternary plot of the normalized repetition rate shift *v*/*δD*_1_ versus the proportions of nonlinear coefficients for *δω* = 0. *κ*_1_ = *κ*_2_ is assumed. The black dashed curve ($g_{11}g_{22} = g_{12}^2$) separates the parameter space into two regions; cross-phase modulation is dominant in the upper region, while self-phase modulation is dominant in the lower region. **b** Plot of the repetition rate shift versus the detuning. *κ*_1_ = *κ*_2_ is assumed. The parameters are *g*_11_ = *g*_12_ and *g*_22_ = 2*g*_12_. The black curve is the analytical result, and the dots are simulated data that use a modified split-step Fourier algorithm adapted to the hybrid system. The inset shows a comparison of the simulated (orange solid line) and analytical (black dashed line) pulse shapes of $|E_1|$ at *δω* = 0. **c** Plot of repetition rate shifts on the mode spectrum. Each line indicates a soliton resonance line with different detunings (negative y-intercept). The parameters are the same as those in (**b**)

For a fixed detuning *δω*, the repetition rate shift *v* depends on the ratios of the nonlinear coefficients *g*_11_, *g*_22_ and *g*_12_. Figure 3a plots the special case of *δω* = 0. When the nonlinearity on the second mode increases, the optical field will shift to the first mode to compensate, leading to an increased overall speed of the pulse, and vice versa. As the repetition rate shift results from the imbalance of self-phase modulations, increasing the proportion of *g*_12_ leads to more stability in the repetition rate, while decreasing the proportion of *g*_12_ allows more tunability. We note that, depending on the nonlinear nature of the resonator material and the mode overlap, the cross-phase modulation may be larger or smaller than the self-phase modulation^52,53^. Moreover, theoretical DS solutions exist for almost all combinations of nonlinear coefficients.

For the tunability of the DS repetition rate, Fig. 3b shows the repetition rate shift as the detuning changes. Near *δω* = −*g*_c_, the repetition rate shift approaches zero, which is consistent with the local KS equivalence argument in the previous section. With a more red-detuned *δω*, the effect of imbalance in the nonlinear coefficients is more apparent, leading to repetition rate changes in the corresponding direction. Simulations of the coupled LLE have also been performed and show that both the simulated pulse shape and the repetition rate shifts agree with the analytical solutions (Fig. 3b). A graphical representation of the repetition rate shift is also shown in Fig. 3c. As an aside, breather-like states^54^ have also been observed in the simulations, but the origin of breathing and whether it behaves in the same way as for KS breathers^55–58^ is not yet fully understood.

Although the discussion so far has focused on pumping at the central mode, it can be readily generalized to off-centre pumping by introducing additional detunings into each of the mode families and shifting the spectral centres of the solutions accordingly. The DS offers a novel and controllable way to tune the repetition rate of the frequency combs. Together with existing nonlinear processes for the resonator, the hybridization-induced shift can be tailored to enhance or suppress the overall repetition rate shift with respect to the pump detuning and may find application in optical frequency division or for pump noise isolation, such as what is performed using quiet point operation^33^.

### Implementation of Dirac solitons

**Fig. 4:**
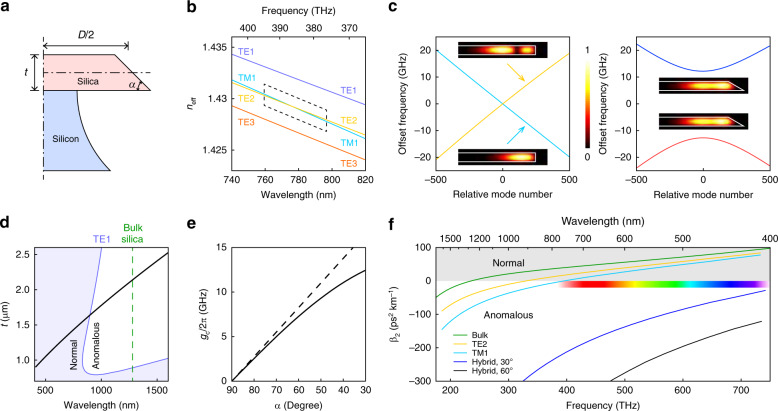
Implementation of mode hybridization. **a** Cross-sectional view of a silica wedge resonator on a silicon pillar (not to scale). The parameters that define the wedge geometry are also shown. **b** Plot of *n*_eff_ for the first four modes (TE1, TM1, TE2, and TE3) versus wavelength (740–820 nm) for a wedge resonator. The parameters are *t* = 1.47 μm and *α* = 90°. **c** Left panel: mode spectrum plot for the boxed region in (**b**). The insets are simulated mode profiles (electric field norm). Right panel: same as left panel but with *α* = 30°. **d** Relationship between *t* and *λ*_*X*_ (black curve). Additionally, the zero-dispersion wavelength of bulk silica (green dashed line) and the zero-dispersion boundary for the TE1 mode (purple curve) are shown. **e** Coupling *g*_c_ versus wedge angle *α* at *λ*_*X*_ = 778 nm. The dashed line is the result from perturbation theory (see ‘Materials and methods’) and is tangent to the *g*_c_ curve at *α* = 90°. **f** Effective GVD *β*_2_ that can be achieved using mode hybridization across the infrared and visible spectra. The parameters are *α* = 30° and *α* = 60°. The dispersion of bulk silica, TE2, and TM1 modes is also shown for comparison. The colour bar shows the approximate colour of light in the visible band

The wedge resonator^59^ is used to induce mode hybridization and Dirac soliton formation. This resonator offers very high-quality factors^60^ and independent control over key parameters during the fabrication process (Fig. 4a). A wedge is entirely characterized by three geometric parameters: the diameter *D*, which depends on the lithographic pattern; the thickness *t*, which depends on the oxidation growth time of the silicon wafer; and the wedge angle *α*, which depends on the adhesion between silica and the photoresist used for patterning. In the following, we will fix the resonator diameter as *D* = 3.2 mm (corresponding to a resonator FSR of ~20 GHz at ~1550 nm), but we note that this can be readily generalized to resonators of other sizes.

For a symmetrical wedge resonator (*α* = 90°), the typical simulated effective refractive index *n*_eff_ versus wavelength is shown in Fig. 4b. At shorter wavelengths, TE1 and TM1 have the highest indices, followed by TE2, TE3 and other high-order modes. Since the electrical fields of the TM modes are along the thickness direction, their indices are more sensitive to changes in the wavelength scale, and the index of TM1 decreases faster than TE2 as the wavelength increases. Eventually, TM1 and TE2 cross, and their relative positions are interchanged at longer wavelengths. However, for *α* = 90°, no hybridization occurs, as the reflection symmetry prohibits interactions between modes of different parities. On the other hand, if we explicitly break the reflection symmetry of the resonator by decreasing the wedge angle (*α* < 90°), the original modes will see an asymmetric change in the refractive index profile, which causes them to couple. Such couplings lift the degeneracy, leading to avoided crossing. The two cases are compared in Fig. 4c, where *n*_eff_ is first converted to the mode number *m* via $m = n_{{\mathrm{eff}}}D\omega _m{\mathrm{/}}(2c)$, where *ω*_*m*_ is the resonance (angular) frequency, and then plotted as offset (angular) frequencies $\omega _{{\mathrm{off}}} = \omega _m - \omega _{\mathrm{X}} - (m - m_{\mathrm{X}})\bar D_1$ versus the relative mode number *m* − *m*_*X*_, where the subscript X indicates the quantity at the degeneracy point. We note that the relative mode number has the same role as the relative wavenumber *k* in the theoretical analyses, except that it is restricted to integer values for periodic boundary conditions.

In view of perturbation theory^61^, the wedge part of the resonator perturbs the underlying symmetrical structure and induces polarization coupling similar to the coupling obtained in directional couplers^62^. Therefore, we expect that the centre wavelength of hybridization *λ*_*X*_ is determined by the thickness *t*, while the wedge angle controls the coupling strength *g*_c_. A plot of *λ*_*X*_ versus *t* is shown as the black curve in Fig. 4d. As *t* is the only geometry scale close to optical wavelengths in the system, we expect that *λ*_*X*_ will scale linearly with *t*, which can be visually verified in the plot. This scaling allows for hybridization to occur at short wavelengths where the dispersion of the original modes (for example, the TE1 mode shown in the figure) is typically normal. A plot of *g*_c_ versus *α* is shown in Fig. 4e. While only a particular wavelength (778 nm) is shown, *g*_c_ depends on the wavelength very weakly, varying less than 5% from wavelengths of 400–1600 nm. The coupling strength scales linearly with *α* near *α* = 90°, which can also be independently verified by first-order perturbation theory (see ‘Materials and methods’), but the coupling effect eventually saturates at shallow wedge angles because mode profiles cannot “squeeze” into the wedge tip as *α* decreases. The calculated GVD *β*_2_ is shown in Fig. 4f, which is related to *D*_2_ via $\beta _2 = - nD_2{\mathrm{/}}(cD_1^2)$. Using suitably designed thicknesses and wedge angles greater than 30°, an anomalous dispersion window can be created all the way down to the blue side of the visible spectrum, where simple geometrical dispersion fails to compensate for normal material dispersion.

### Demonstration of Dirac solitons

**Fig. 5:**
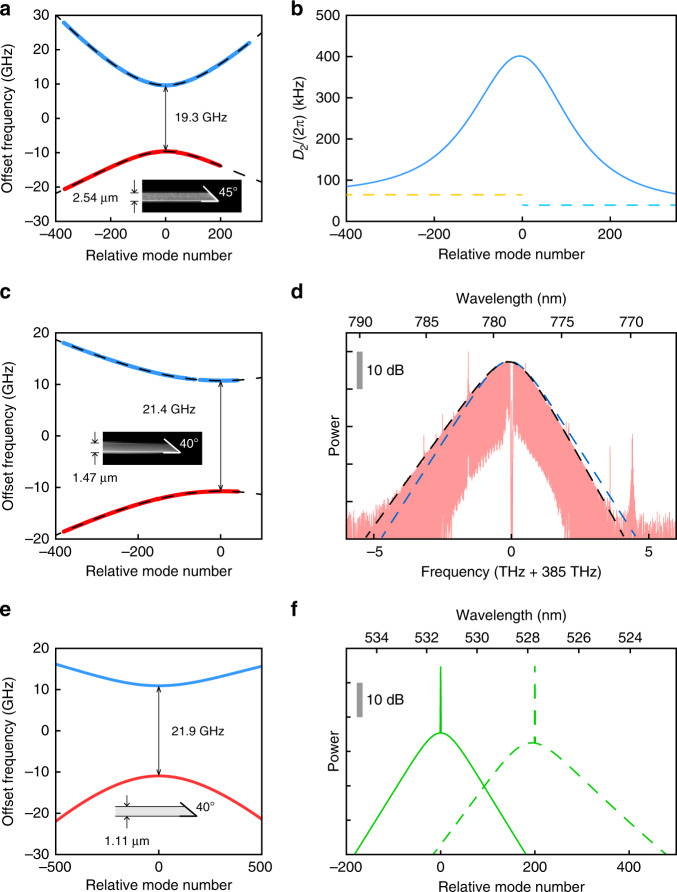
Demonstrations of mode hybridization and DS generation. **a** Measured mode spectrum for a wedge resonator with mode hybridization at approximately 1550 nm. The inset shows the geometry of the resonator. **b** Second-order dispersion of the upper branch derived from the fit in (**a**). The two dashed lines indicate the second-order dispersion of the underlying TE2 (left) and TM1 (right) modes without crossing. **c** Measured mode spectrum for a wedge resonator with mode hybridization at ~778 nm. The inset shows the geometry of the resonator. **d** Optical spectrum of a DS at 778 nm generated in a wedge resonator. The blue dashed line shows the sech^2^ fit, and the black dashed line shows the DS fit. **e** Simulated mode spectrum for a wedge resonator with mode hybridization at approximately 532 nm. The inset shows the geometry of the resonator. **f** Simulated spectrum of a DS at 532 nm generated in the wedge resonator in (**e**). The solid line shows the spectrum when the system is pumped at the crossing centre, while the dashed line shows the spectrum when the system is pumped 200 modes away from the crossing centre. The detuning from the upper branch is fixed at 100 MHz. The mode quality factors are taken as 20 × 10^6^ and are critically coupled. The pump powers are taken as 250 mW, and the polarization is matched to the mode in the upper branch being pumped

Guided by these design principles, devices that target 1550 nm and 778 nm as their hybridization wavelengths were fabricated. The mode spectra are measured for each device using a tuneable laser and a calibrated Mach-Zehnder interferometer^8^. As expected, each of the devices shows a pair of modes with hyperbolic dispersion and large curvatures (Fig. 5a, c). The local *D*_2_ of the anomalous branch can be fit to give *D*_2_ = 2*π* × 401 kHz (1550 nm) (Fig. 5b) and *D*_2_ = 2*π* × 132 kHz (778 nm), corresponding to *β*_2_ = −790 ps^2^ km^−1^ and *β*_2_ = −255 ps^2^ km^−1^, respectively, which are orders of magnitude larger than the mode intrinsic GVD without hybridization.

Finally, to demonstrate the existence of DSs in wedge resonators with hybridized modes, we generated solitons at 778 nm. The detailed experimental setup and measurement procedures can be found elsewhere^36^. The optical spectrum of the soliton is shown in Fig. 5. A direct sech^2^ fit to the spectrum reveals that the frequency components are highly asymmetric around the spectrum centre, which results from the high-order dispersions of the mode and is a typical feature for DSs not being pumped in the crossing centre. Indeed, the analytical DS solution derived here provides a good fit to the measured spectrum, further confirming the above theoretical results and their applicability for soliton modelling in microresonators. The detuning relative to the upper branch can be inferred from the fitting and is approximately 90 MHz, which is less than 5% of the band gap. We did not attempt to increase the detuning further due to pump power limitations. As an aside, we believe that a similar 780-nm soliton generated in^36^, using the same resonator but under different pumping conditions, can also be classified as a DS.

To further validate the feasibility of DSs in the visible band, we also performed simulations of a DS near 532 nm. A resonator design is shown in Fig. 5e together with its simulated mode dispersions, and solitons can be found in the resonator with different pumping positions (Fig. 5f). The soliton at the crossing centre has a more symmetric spectrum, while the soliton far away from the crossing centre has a more skewed spectrum, as expected.

## Discussion

Critical to the generation of solitons in microresonators is the control of mode dispersion. Bright soliton formation requires anomalous dispersion. The curvature of a circular resonator contributes to normal dispersion, shifting the zero-dispersion wavelength towards longer wavelengths as the resonator size decreases. Both this and material dispersion have been managed over a range of wavelengths by using geometrical dispersion introduced through optical waveguide confinement^1^. However, normal dispersion of bulk dielectric materials increases towards shorter wavelengths making visible soliton generation in dielectric microresonators difficult. And the offset of this normal dispersion through waveguide confinement increases unwanted scattering loss, which degrades the resonator *Q* factor and increases the pumping power (i.e., the comb threshold power varies inversely quadratically with the *Q* factor^8,63^). While the use of intra-cavity nonlinear optical processes such as second- or third-harmonic generation and sum-frequency generation can provide a way to bypass normal dispersion for comb generation in the visible band^13,64–67^, managing dispersion by mode coupling provides an alternative approach that can avoid waveguide confinement loss^36,37,68–72^. The practical management of dispersion for soliton formation at the edge of the visible band^36,37^ has been possible using Dirac solitons, and they can provide a way to further extend operation well into the visible region.

In summary, we demonstrated the peculiar characteristics of the Dirac soliton in microresonators by solving the corresponding conservative coupled LLE non-perturbatively and using the exact solution as a soliton ansatz for the hybrid system. The balance between nonlinearity and coupling provides a new viewpoint on the soliton and opens up further directions for the study of soliton dynamics. From an experimental viewpoint, generating DSs in resonator platforms is straightforward, but it might be challenging to observe some of their more unusual properties at large detunings. Decreasing the linear coupling (and thus the band gap) is beneficial for pushing the soliton deeper into the band gap, and tuning the pumping polarization to match the soliton decreases the pumping power requirements. We believe the formalism described here can be readily generalized to other systems, including Dirac solitons in waveguides, solitons on ordinary (non-polarization) mode crossings^33^ and counter-propagating solitons with moderate coupling^73^.

## Materials and methods

### Solving the conservative coupled LLE

We copy the conservative coupled LLE here for convenience:

$$\begin{array}{l}\frac{{\partial E_1}}{{\partial t}} = - i\delta \omega E_1 + ig_cE_2 - \delta D_1\frac{{\partial E_1}}{{\partial \theta }} + i(g_{11}|E_1|^2E_1 + g_{12}|E_2|^2E_1)\\ \frac{{\partial E_2}}{{\partial t}} = - i\delta \omega E_2 + ig_cE_1 + \delta D_1\frac{{\partial E_2}}{{\partial \theta }} + i(g_{22}|E_2|^2E_2 + g_{12}|E_1|^2E_2)\end{array}$$

We seek soliton solutions in the form of *E*_1,2_(*θ* − *vt*), where *v* is the repetition rate shift in the symmetric co-moving frame, which reduces the partial differential equations to ordinary differential equations:

$$\begin{array}{c}(\delta D_1 - v)\partial _\theta E_1 = - i\delta \omega E_1 + ig_cE_2 + i(g_{11}|E_1|^2E_1 + g_{12}|E_2|^2E_1)\\ - (\delta D_1 + v)\partial _\theta E_2 = - i\delta \omega E_2 + ig_cE_1 + i(g_{22}|E_2|^2E_2 + g_{12}|E_1|^2E_2)\end{array}$$

Continuous symmetries of the system result in conservation laws^74^, which can reduce the dimensions of the system. As the system is conservative, we expect the equations will have a Hamiltonian structure. Indeed, the following quantity is conserved when *θ* is viewed as an evolution coordinate^46^:

$$\begin{array}{c}\bar H = - \delta \omega (|E_1|^2 + |E_2|^2) + g_{\mathrm{c}}(E_1^ \ast E_2 + E_2^ \ast E_1)\\ + \frac{1}{2}\left( {g_{11}|E_1|^4 + g_{22}|E_2|^4 + 2g_{12}|E_1|^2|E_2|^2} \right)\end{array}$$

The conservation of $\bar H$ can be verified by rewriting $(\delta D_1 - v)\partial _\theta E_1 = i\partial _{E_1^ \ast }\bar H$ and $- (\delta D_1 + v)\partial _\theta E_2 = i\partial _{E_2^ \ast }\bar H$. Another quantity that is conserved is the photon number flow along the *θ*-axis:

$$\bar N = (\delta D_1 - v)|E_1|^2 - (\delta D_1 + v)|E_2|^2$$

The conservation of $\bar N$ can be verified by observing that all the nonlinear terms do not change the individual numbers of particles, while the coupling terms do not change the total number of particles.

For soliton solutions, these two conserved quantities can be determined as $\bar H = \bar N = 0$ since the solution should vanish exponentially as *θ* → ∞ without periodic boundary conditions. This determination leads to the following amplitude-phase parametrization of the solutions:

$$\begin{array}{c}E_1 = \frac{1}{{\sqrt {\delta D_1 - v} }}\psi {\mathrm{exp}}(i\chi _1),E_2 = - \frac{1}{{\sqrt {\delta D_1 + v} }}\psi {\mathrm{exp}}(i\chi _2)\\ \psi = \sqrt {\delta D_1 - v} |E_1| = \sqrt {\delta D_1 + v} |E_2|,\chi _{1,2} = \frac{1}{{2i}}{\mathrm{ln}}\frac{{E_{1,2}}}{{E_{1,2}^ \ast }}\end{array}$$

which automatically satisfies the $\bar N$ conservation (the negative sign is added for later convenience). The $\bar H$ conservation reads as:

$$\begin{array}{c} - \frac{{2\delta D_1\delta \omega }}{{\delta D_1^2 - v^2}}\psi ^2 - \frac{{2g_{\mathrm{c}}}}{{\sqrt {\delta D_1^2 - v^2} }}\psi ^2{\mathrm{cos}}(\chi _2 - \chi _1)\\ + \left[ {\frac{{g_{11}}}{{2(\delta D_1 - v)^2}} + \frac{{g_{22}}}{{2(\delta D_1 + v)^2}} + \frac{{g_{12}}}{{\delta D_1^2 - v^2}}} \right]\psi ^4\\ = 0\end{array}$$

from which the cosine of the phase difference *χ*_2_ − *χ*_1_ can be solved as:

$${\mathrm{cos}}(\chi _2 - \chi _1) = \frac{{G\psi ^2 - 2\delta D_1\delta \omega }}{{2g_c\sqrt {\delta D_1^2 - v^2} }}$$

where for convenience, we defined a combined nonlinear coefficient:

$$G = \frac{{\delta D_1 + v}}{{\delta D_1 - v}}\frac{{g_{11}}}{2} + \frac{{\delta D_1 - v}}{{\delta D_1 + v}}\frac{{g_{22}}}{2} + g_{12}$$

Turning back to the original equations of evolution along *θ*, we substitute *E*_1,2_ with the parametrization and split the real and imaginary parts:

$$\begin{array}{c}\frac{{\partial \psi ^2}}{{\partial \theta }} = \frac{{2g_{\mathrm{c}}}}{{\sqrt {\delta D_1^2 - v^2} }}\psi ^2{\mathrm{sin}}(\chi _2 - \chi _1)\\ \frac{{\partial \chi _1}}{{\partial \theta }} = - \frac{{\delta \omega }}{{\delta D_1 - v}} - \frac{{g_{\mathrm{c}}}}{{\sqrt {\delta D_1^2 - v^2} }}{\mathrm{cos}}(\chi _2 - \chi _1) + \left( {\frac{{g_{11}}}{{(\delta D_1 - v)^2}} + \frac{{g_{12}}}{{\delta D_1^2 - v^2}}} \right)\psi ^2\\ - \frac{{\partial \chi _2}}{{\partial \theta }} = - \frac{{\delta \omega }}{{\delta D_1 + v}} - \frac{{g_{\mathrm{c}}}}{{\sqrt {\delta D_1^2 - v^2} }}{\mathrm{cos}}(\chi _2 - \chi _1) + \left( {\frac{{g_{22}}}{{(\delta D_1 + v)^2}} + \frac{{g_{12}}}{{\delta D_1^2 - v^2}}} \right)\psi ^2\end{array}$$

For the differential equation for *ψ*^2^, expressing sin(*χ*_2_ − *χ*_1_) in terms of *ψ*^2^ gives:

$$\frac{{\partial \psi ^2}}{{\partial \theta }} = \pm \frac{{2g_{\mathrm{c}}}}{{\sqrt {\delta D_1^2 - v^2} }}\psi ^2\sqrt {1 - \left( {\frac{{G\psi ^2 - 2\delta D_1\delta \omega }}{{2g_{\mathrm{c}}\sqrt {\delta D_1^2 - v^2} }}} \right)^2}$$

which can be integrated (with the boundary condition *ψ*^2^ → 0 as *θ* → ∞) in terms of elementary functions:

$$\psi ^2 = \frac{{g_{\mathrm{c}}\sqrt {\delta D_1^2 - v^2} }}{G}\frac{{2(1 - \tilde \xi ^2)}}{{{\mathrm{cosh}}\left( {2\sqrt {1 - \tilde \xi ^2} \tilde \theta } \right) - \tilde \xi }}$$

where the pulse centre is chosen as *θ* = 0 without loss of generality and the reduced detuning and coordinate are defined as:

$$\begin{array}{l}\tilde \xi = \frac{{\delta D_1}}{{\sqrt {\delta D_1^2 - v^2} }}\frac{{\delta \omega }}{{g_{\mathrm{c}}}}\\ \tilde \theta = \frac{{g_{\mathrm{c}}}}{{\sqrt {\delta D_1^2 - v^2} }}\theta \end{array}$$

As an aside, we also obtain that:

$${\mathrm{cos}}(\chi _2 - \chi _1) = \frac{{1 - \tilde \xi {\mathrm{cosh}}\left( {2\sqrt {1 - \tilde \xi ^2} \tilde \theta } \right)}}{{{\mathrm{cosh}}\left( {2\sqrt {1 - \tilde \xi ^2} \tilde \theta } \right) - \tilde \xi }}$$

The differential equation for *χ*_1,2_ can be integrated after substitution of the above solution for *ψ*^2^ and cos(*χ*_2_ − *χ*_1_). Because the equation has global phase symmetry ($E_{1,2} \to e^{i\phi }E_{1,2}$, where *ϕ* is an arbitrary constant phase), we can fix *χ*_1_(*θ* = 0) = 0, which also forces *χ*_2_ = 0 through ${\mathrm{cos}}(\chi _1 - \chi _2)|_{\theta = 0} = 1$. We obtain:

$$\begin{array}{l}\chi _1 = - \frac{v}{{\delta D_1}}\tilde \xi \tilde \theta + \left[ {\frac{1}{G}\left( {\frac{{\delta D_1 + v}}{{\delta D_1 - v}}g_{11} - \frac{{\delta D_1 - v}}{{\delta D_1 + v}}g_{22}} \right) + 1} \right]{\mathrm{arctan}}\left[ {\sqrt {\frac{{1 + \tilde \xi }}{{1 - \tilde \xi }}} {\mathrm{tanh}}\left( {\sqrt {1 - \tilde \xi ^2} \tilde \theta } \right)} \right]\\ \chi _2 = - \frac{v}{{\delta D_1}}\tilde \xi \tilde \theta - \left[ {\frac{1}{G}\left( {\frac{{\delta D_1 - v}}{{\delta D_1 + v}}g_{22} - \frac{{\delta D_1 + v}}{{\delta D_1 - v}}g_{11}} \right) + 1} \right]{\mathrm{arctan}}\left[ {\sqrt {\frac{{1 + \tilde \xi }}{{1 - \tilde \xi }}} {\mathrm{tanh}}\left( {\sqrt {1 - \tilde \xi ^2} \tilde \theta } \right)} \right]\end{array}$$

With these results, the soliton solutions can be expressed as:

$$\begin{array}{c}E_1 = + \sqrt {\frac{{2g_{\mathrm{c}}}}{G}} \sqrt {1 - \tilde \xi ^2} (\frac{{\delta D_1 + v}}{{\delta D_1 - v}})^{1/4}\frac{{\left[ {{\mathrm{cosh}}\left( {2\sqrt {1 - \tilde \xi ^2} \tilde \theta } \right) - \tilde \xi } \right]^{\gamma /2}}}{{\left[ {\sqrt {1 - \tilde \xi } {\mathrm{cosh}}\left( {\sqrt {1 - \tilde \xi ^2} \tilde \theta } \right) - i\sqrt {1 + \tilde \xi } {\mathrm{sinh}}\left( {\sqrt {1 - \tilde \xi ^2} \tilde \theta } \right)} \right]^{1 + \gamma }}}\\ {\mathrm{exp}}\left( { - i\frac{v}{{\delta D_1}}\tilde \xi \tilde \theta } \right)\\ E_2 = - \sqrt {\frac{{2g_{\mathrm{c}}}}{G}} \sqrt {1 - \tilde \xi ^2} (\frac{{\delta D_1 - v}}{{\delta D_1 + v}})^{1/4}\frac{{\left[ {{\mathrm{cosh}}\left( {2\sqrt {1 - \tilde \xi ^2} \tilde \theta } \right) - \tilde \xi } \right]^{ - \gamma /2}}}{{\left[ {\sqrt {1 - \tilde \xi } {\mathrm{cosh}}\left( {\sqrt {1 - \tilde \xi ^2} \tilde \theta } \right) + i\sqrt {1 + \tilde \xi } {\mathrm{sinh}}\left( {\sqrt {1 - \tilde \xi ^2} \tilde \theta } \right)} \right]^{1 - \gamma }}}\\ {\mathrm{exp}}\left( { - i\frac{v}{{\delta D_1}}\tilde \xi \tilde \theta } \right)\end{array}$$

where we introduce the phase exponent:

$$\gamma = \frac{1}{G}\left( {\frac{{\delta D_1 + v}}{{\delta D_1 - v}}g_{11} - \frac{{\delta D_1 - v}}{{\delta D_1 + v}}g_{22}} \right)$$

Although we have not been very rigorous for multivalued functions encountered in the calculations, direct substitution shows that the *E*_1,2_ obtained above is indeed a solution to the original conservative LLE when principal branches are used.

### Resonance line and the band gap

The general bright soliton solution includes the square root of $1 - \tilde \xi ^2$, which requires that $|\tilde \xi |\, < \,{1}$. Expanded with resonator parameters, this gives:

$$|\delta \omega | \le \frac{{\sqrt {\delta D_1^2 - v^2} }}{{\delta D_1}}g_{\mathrm{c}}$$

For a fixed *v*, the inequality gives the detuning range where the solution is well-defined. A quick plot of the range (Fig. 2b) shows that the boundaries are tangent to the mode spectrum curves. Indeed, using coupled mode theory, the frequencies can be described as:

$$\omega _ \pm = \mp \sqrt {\delta D_1^2k^2 + g_{\mathrm{c}}^2}$$

where *ω*_+_ (*ω*_−_) is the eigenfrequency for the lower symmetric branch (upper anti-symmetric branch) and *k* is the wavenumber. The tangent lines for the upper branch with slope *v* satisfy:

$$v = \frac{{\partial \omega _ - }}{{\partial k}} = \frac{{\delta D_1^2k}}{{\sqrt {\delta D_1^2k^2 + g_{\mathrm{c}}^2} }}$$

Eliminating *k* recovers the previous boundaries. Thus, the soliton resonance lines can stay only in the band gap and cannot cut through the band curves.

We note that in dissipative cases, *v* is not fixed but depends on the pumping details (as discussed in the main text), so this point should not be understood as a limitation on detuning when pumping the soliton. Instead, the detuning range should be determined from the momentum constraints imposed on the soliton at fixed *k* (the longitudinal mode being pumped).

### Reduction of a DS to a KS

Following the above discussions on resonance lines, we focus on the case in which $\tilde \xi \to - 1^ +$, where the resonance line is almost tangent to the upper branch of the mode spectrum. Taking the limits and expanding the reduced quantities results in:

$$\begin{array}{c}E_{1,2} = \pm \sqrt {\frac{{2g_{\mathrm{c}}}}{G}} \sqrt {1 + \tilde \xi } (\frac{{\delta D_1 \pm v}}{{\delta D_1 \mp v}})^{1/4}{\mathrm{sech}}\left( {\sqrt {2(1 + \tilde \xi )} \tilde \theta } \right){\mathrm{exp}}\left( {i\frac{v}{{\delta D_1}}\tilde \theta } \right)\\ E_{1,2} = \pm \sqrt {\frac{{2\delta D_1(\delta \omega - \delta \omega _{{\mathrm{min}}})}}{{G(\delta D_1 \mp v)}}} {\mathrm{sech}}\left( {\sqrt {2(\delta \omega - \delta \omega _{{\mathrm{min}}})} \sqrt {\frac{{g_{\mathrm{c}}\delta D_1}}{{(\delta D_1^2 - v^2)^{3/2}}}} \theta } \right)\\ {\mathrm{exp}}\left( {i\frac{{g_{\mathrm{c}}v}}{{\delta D_1\sqrt {\delta D_1^2 - v^2} }}\theta } \right)\end{array}$$

where $\delta \omega _{{\mathrm{min}}} = - g_{\mathrm{c}}\sqrt {\delta D_1^2 - v^2} {\mathrm{/}}\delta D_1$. The hyperbolic secant form is now apparent, and to complete the reduction, we explicitly calculate the local quantities of the mode spectrum.

When the resonance line is tangent to the mode spectrum, the wavenumber *k* can be solved from the previous section:

$$k = \frac{{g_{\mathrm{c}}v}}{{\delta D_1\sqrt {\delta D_1^2 - v^2} }}$$

which matches the exponential term. The minimum detuning that can be achieved at this particular *m* also matches *δω*_min_. The local second-order dispersion is given by:

$$\frac{{\partial ^2\omega _ - }}{{\partial k^2}} = \frac{{g_{\mathrm{c}}^2\delta D_1^2}}{{(\delta D_1^2k^2 + g_{\mathrm{c}}^2)^{3/2}}} = \frac{{(\delta D_1^2 - v^2)^{3/2}}}{{g_c\delta D_1}}$$

where we have eliminated *k* using *v* and it matches the dispersion term. The mode composition can be found using coupled mode theory and is given by:

$$\frac{{E_1}}{{E_2}} = - \frac{{\omega _ - + \delta D_1k}}{{g_{\mathrm{c}}}} = \frac{{\sqrt {\delta D_1 + v} }}{{\sqrt {\delta D_1 - v} }}$$

which agrees with the prefactors in *E*_1,2_ and is also consistent with the conservation of $\bar N$. Finally, the effective nonlinear coefficient $G(\delta D_1^2 - v^2){\mathrm{/}}(2\delta D_1^2)$ can be calculated as a weighted average of the nonlinear coefficients, the weight being the power proportions on each mode derived above. It matches the nonlinear coefficient except for the extra factor $(\delta D_1 \pm v){\mathrm{/}}(2\delta D_1)$, which is the power ratio of each mode component to the total power and is a result of expressing the solution using components rather than the hybridized field.

To complete the discussion of reducing a DS to a KS, we also present a perturbative approach that is explicitly based on the hybridized field. We begin by defining the following auxiliary fields:

$$\begin{array}{l}\psi _ - = \left( {\sqrt {\frac{{\delta D_1 - v}}{{2\delta D_1}}} E_1 - \sqrt {\frac{{\delta D_1 + v}}{{2\delta D_1}}} E_2} \right){\mathrm{exp}}\left( {i\frac{v}{{\delta D_1}}\tilde \xi \tilde \theta } \right)\\ \psi _ + = \left( {\sqrt {\frac{{\delta D_1 - v}}{{2\delta D_1}}} E_1 + \sqrt {\frac{{\delta D_1 + v}}{{2\delta D_1}}} E_2} \right){\mathrm{exp}}\left( {i\frac{v}{{\delta D_1}}\tilde \xi \tilde \theta } \right)\end{array}$$

We note that while the *ψ*_+_ component is the normalized linear eigenstate of the lower branch at the wavenumber corresponding to *v*, the *ψ*_−_ term defined here is, in general, not the eigenstate of the upper branch, and *ψ*_−_ and *ψ*_+_ are not orthogonal (although in the special case *ψ*_+_ = 0, *ψ*_−_ becomes proportional to the true field amplitude). Rewriting the conservative coupled LLE in terms of *ψ*_±_ results in:

$$\begin{array}{l}\frac{{\partial \psi _ + }}{{\partial \tilde \theta }} = - i(1 + \tilde \xi )\psi _ - + \frac{{i\delta D_1}}{{2g_c\sqrt {\delta D_1^2 - v^2} }}\left[ {\frac{{\delta D_1 + v}}{{\delta D_1 - v}}\frac{{g_{11}}}{2}|\psi _ + + \psi _ - |^2(\psi _ + + \psi _ - )} \right.\\ - g_{12}(\psi _ + ^2 - \psi _ - ^2)\psi _ - ^ \ast \\ \left. { - \frac{{\delta D_1 - v}}{{\delta D_1 + v}}\frac{{g_{22}}}{2}|\psi _ + - \psi _ - |^2(\psi _ + - \psi _ - )} \right]\\ \frac{{\partial \psi _ - }}{{\partial \tilde \theta }} = i(1 - \tilde \xi )\psi _ + + \frac{{i\delta D_1}}{{2g_c\sqrt {\delta D_1^2 - v^2} }}\left[ {\frac{{\delta D_1 + v}}{{\delta D_1 - v}}\frac{{g_{11}}}{2}|\psi _ + + \psi _ - |^2(\psi _ + + \psi _ - )} \right.\\ + g_{12}(\psi _ + ^2 - \psi _ - ^2)\psi _ + ^ \ast \\ \left. { + \frac{{\delta D_1 - v}}{{\delta D_1 + v}}\frac{{g_{22}}}{2}|\psi _ + - \psi _ - |^2(\psi _ + - \psi _ - )} \right]\end{array}$$

where we have substituted *δω* and *θ* with $\tilde \xi$ and $\tilde \theta$, respectively, for later convenience. Based on the structure of the above equation, we seek the following solution near $\tilde \xi \to - 1^ +$:

$$\psi _ - \sim O(1 + \tilde \xi )^{1/2},\psi _ + \sim O(1 + \tilde \xi )$$

Keeping the lowest-order terms gives:

$$\begin{array}{l}\frac{{\partial \psi _ + }}{{\partial \tilde \theta }} = - i(1 + \tilde \xi )\psi _ - + \frac{{i\delta D_1}}{{2g_c\sqrt {\delta D_1^2 - v^2} }}G|\psi _ - |^2\psi _ - \\ \frac{{\partial \psi _ - }}{{\partial \tilde \theta }} = 2i\psi _ + \end{array}$$

Combining gives:

$$\frac{1}{2}\frac{{\partial ^2\psi _ - }}{{\partial \tilde \theta ^2}} - (1 + \tilde \xi )\psi _ - + \frac{{\delta D_1}}{{2g_c\sqrt {\delta D_1^2 - v^2} }}G|\psi _ - |^2\psi _ - = 0$$

which is the steady-state single-mode LLE and its solution is the same as the limit of *E*_1,2_ as derived above.

### Repetition rate shifts in the DS

We copy the dissipative coupled LLE here for convenience:

$$\begin{array}{c}\frac{{\partial E_1}}{{\partial t}} = - i\delta \omega E_1 + ig_cE_2 - \delta D_1\frac{{\partial E_1}}{{\partial \theta }} + i(g_{11}|E_1|^2E_1 + g_{12}|E_2|^2E_1)\\ - \frac{{\kappa _1}}{2}E_1 + f_1\\ \frac{{\partial E_2}}{{\partial t}} = - i\delta \omega E_2 + ig_cE_1 + \delta D_1\frac{{\partial E_2}}{{\partial \theta }} + i(g_{22}|E_2|^2E_2 + g_{12}|E_1|^2E_2)\\ - \frac{{\kappa _2}}{2}E_2 + f_2\end{array}$$

We define the following momentum integral in the hybrid system:

$$P = {\int} {\left[ {E_1^ \ast \left( { - i\frac{{\partial E_1}}{{\partial \theta }}} \right) + E_2^ \ast \left( { - i\frac{{\partial E_2}}{{\partial \theta }}} \right)} \right]} d\theta$$

For a steady-state solution, *P* should be a constant in time. We thus calculate the first derivative of *P* with respect to *t*:

$$0 = \frac{{\partial P}}{{\partial t}} = {\int} {\left( { - i\frac{{\partial E_1^ \ast }}{{\partial t}}\frac{{\partial E_1}}{{\partial \theta }} + i\frac{{\partial E_1}}{{\partial t}}\frac{{\partial E_1^ \ast }}{{\partial \theta }} - i\frac{{\partial E_2^ \ast }}{{\partial t}}\frac{{\partial E_2}}{{\partial \theta }} + i\frac{{\partial E_2}}{{\partial t}}\frac{{\partial E_2^ \ast }}{{\partial \theta }}} \right)} d\theta$$

where we have used integration by parts to move the spatial derivatives to the conjugated field. After plugging the equations of motion into the integral, all the conservative terms cancel each other out, and the pumping terms vanish by integration by parts. We are left with:

$$\frac{{\kappa _1}}{2}{\int} {\left( {iE_1^ \ast \frac{{\partial E_1}}{{\partial \theta }} - iE_1\frac{{\partial E_1^ \ast }}{{\partial \theta }}} \right)} d\theta + \frac{{\kappa _2}}{2}{\int} {\left( {iE_2^ \ast \frac{{\partial E_2}}{{\partial \theta }} - iE_2\frac{{\partial E_2^ \ast }}{{\partial \theta }}} \right)} d\theta = 0$$

Rewriting the above equation using arguments gives:

$$\kappa _1{\int} {|E_1|^2\frac{{\partial {\mathrm{arg}}E_1}}{{\partial \theta }}d\theta } + \kappa _2{\int} {|E_2|^2\frac{{\partial {\mathrm{arg}}E_2}}{{\partial \theta }}d\theta = 0}$$

To proceed further, we take the soliton ansatz as the exact solution of the DS derived earlier. In this case, the integration can be carried out analytically:

$${\int} {|E_{1,2}|^2} \frac{{\partial {\mathrm{arg}}E_{1,2}}}{{\partial \theta }}d\theta = \frac{{2g_c}}{G}\sqrt {\frac{{\delta D_1 \pm v}}{{\delta D_1 \mp v}}} \left[ {\left( { - \frac{v}{{\delta D_1}} + \gamma \pm 1} \right)(\pi - {\mathrm{arccos}}\tilde \xi )\tilde \xi + (\gamma \pm 1)\sqrt {1 - \tilde \xi ^2} } \right]$$

All the quantities can be explicitly expressed in *v*, and the resulting equation can be solved numerically. In the special case of *κ*_1_ = *κ*_2_ and *δω* = 0, we have $\tilde \xi = 0$ independent of *v*, and the criterion is greatly simplified:

$$\frac{v}{{\delta D_1}} = - \gamma$$

Expanding *γ* gives a cubic equation in *v* and is used in the plot of Fig. 3a.

### First-order perturbation calculation of mode coupling in wedge resonators

Here, using first-order degeneracy perturbation theory and the integral form of the propagation constant, we derive the mode coupling in wedge resonators as an overlap integral of the unperturbed modes.

**Fig. 6:**
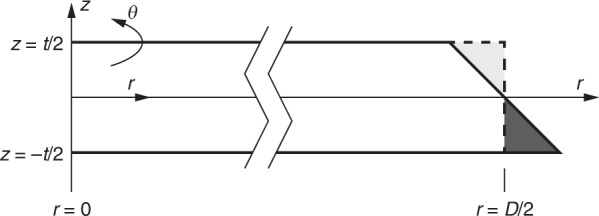
Illustration of the perturbation induced by the wedge angle in the wedge resonator. The light grey area indicates the dielectric removed compared to a symmetric resonator, while the dark grey area indicates the dielectric added. The cylindrical coordinates used to describe the resonator are also shown

For a circular waveguide, the angular momentum number (angular propagation constant) of a mode can be expressed as^61^:

$$m = \frac{{\omega _0}}{{2c}}\frac{{{\int} {\left[ {n^2(E_r^ \ast E_r + E_z^ \ast E_z - E_\theta ^ \ast E_\theta ) + c^2(B_r^ \ast B_r + B_z^ \ast B_z - B_\theta ^ \ast B_\theta )} \right]} rdrdz}}{{{\int} {c(E_rB_z^ \ast - E_zB_r^ \ast )drdz} }}$$

where *ω*_0_ is the angular frequency of the light and *E*_*r*_, *E*_*z*_, *E*_θ_ (*B*_*r*_, *B*_*z*_, *B*_θ_) are the mode electric field (magnetic flux density) components (the coordinate system in use is shown in Fig. 6). The linear propagation of a field (with fixed *ω*_0_) is described by $dE_1/d\theta = imE_1$, where *E*_1_ is the field amplitude at different angular positions. If the mode profile in another waveguide with a slightly different shape is nearly identical to the current waveguide, which is usually true up to the first-order of the geometry differences, then the same integral can be used to calculate the propagation constant using the known field profile and the perturbed refractive index profile.

For a pair of nearly degenerate modes, the propagation constant generalizes into a matrix:

$$\frac{d}{{d\theta }}\left( {\begin{array}{*{20}{c}} {E_1} \\ {E_2} \end{array}} \right) = i\left( {\begin{array}{*{20}{c}} {m_{11}} & {m_{12}} \\ {m_{21}} & {m_{22}} \end{array}} \right)\left( {\begin{array}{*{20}{c}} {E_1} \\ {E_2} \end{array}} \right)$$

$$m_{ij} = \frac{{\omega _0}}{{2c}}\frac{{{\int} {\left[ {n^2(E_{r,i}^ \ast E_{r,j} + E_{z,i}^ \ast E_{z,j} - E_{\theta ,i}^ \ast E_{\theta ,j}) + c^2(B_{r,i}^ \ast B_{r,j} + B_{z,i}^ \ast B_{z,j} - B_{\theta ,i}^ \ast B_{\theta ,j})} \right]} rdrdz}}{{\sqrt {{\int} {c(E_{r,i}B_{z,i}^ \ast - E_{z,i}B_{r,i}^ \ast )drdz} } \sqrt {{\int} {c(E_{r,j}B_{z,j}^ \ast - E_{z,j}B_{r,j}^ \ast )drdz} } }}$$

The off-diagonal elements *m*_12_ and *m*_21_ have an overlap integral structure and are proportional to *g*_c_. Since the modes are orthogonal in the original waveguide, only the changes in refractive index induce coupling:

$$m_{ij} = \frac{{\omega _0}}{{2c}}\frac{{{\int} {\Delta (n^2)(E_{r,i}^ \ast E_{r,j} + E_{z,i}^ \ast E_{z,j} - E_{\theta ,i}^ \ast E_{\theta ,j})rdrdz} }}{{\sqrt {{\int} {c(E_{r,i}B_{z,i}^ \ast - E_{z,i}B_{r,i}^ \ast )drdz} } \sqrt {{\int} {c(E_{r,j}B_{z,j}^ \ast - E_{z,j}B_{r,j}^ \ast )drdz} } }}$$

where Δ(*n*^2^) is the change in *n*^2^ of the perturbed waveguide compared to the original waveguide.

The introduction of the wedge angle adds a dielectric triangle to the lower-right part and subtracts a dielectric triangle to the upper-right part (Fig. 6). As these are the only areas in which the refractive index changes, the overlap integral is effectively restricted to the triangles. If *π*/2 − *α* is small, we can further replace all the fields by their values on the vertical boundary of the wedge. This replacement results in:

$$m_{12} \approx \frac{{\omega _0}}{{2c}}\left( {n_{\mathrm{M}}^2 - 1} \right)\frac{{ - {\int}_{ - t/2}^{t/2} {\left( {E_{r,1}^ \ast E_{r,2} + E_{z,1}^ \ast E_{z,2} - E_{\theta ,1}^ \ast E_{\theta ,2}} \right)} (D/2)(\pi /2 - \alpha )zdz}}{{\sqrt {{\int} {c(E_{r,1}B_{z,1}^ \ast - E_{z,1}B_{r,1}^ \ast )drdz} } \sqrt {{\int} {c(E_{r,2}B_{z,2}^ \ast - E_{z,2}B_{r,2}^ \ast )drdz} } }}$$

where *n*_M_ is the dielectric index. The integral can be further reduced by symmetry, using $E_{r,1}(z) = E_{r,1}( - z)$, $E_{z,1}(z) = - E_{z,1}( - z)$ and $E_{\theta ,1}(z) = E_{\theta ,1}( - z)$ for the TE mode and $E_{r,2}(z) = - E_{r,2}( - z)$, $E_{z,2}(z) = E_{z,2}( - z)$ and $E_{\theta ,2}(z) = - E_{\theta ,2}( - z)$ for the TM mode. This process reduces the integration limits by half:

$$m_{12} \approx \frac{{\omega _0}}{c}\frac{D}{2}(n_{\mathrm{M}}^2 - 1)\frac{{ - {\int}_0^{t/2} {[(n_{\mathrm{M}}^2 + 1)D_{r,1}^ \ast D_{r,2}{\mathrm{/}}(2n_{\mathrm{M}}^2\varepsilon _0^2) + E_{z,1}^ \ast E_{z,2} - E_{\theta ,1}^ \ast E_{\theta ,2}]_{r = D/2}} zdz}}{{\sqrt {{\int} {c(E_{r,1}B_{z,1}^ \ast - E_{z,1}B_{r,1}^ \ast )drdz} } \sqrt {{\int} {c(E_{r,2}B_{z,2}^ \ast - E_{z,2}B_{r,2}^ \ast )drdz} } }}(\frac{\pi }{2} - \alpha )$$

where the radial electric field is replaced by the electric displacement field *D*_*r*_ to prevent ambiguities across the dielectric boundary; *ε*_0_ is the vacuum permittivity.

Finally, *m*_12_ can be converted to *g*_c_ in the same way that the effective index is converted to the mode spectrum:

$$g_{\mathrm{c}} = \frac{{2c}}{{n_{{\mathrm{eff}}}D}}|m_{12}| \approx \frac{{\omega _0}}{{n_{{\mathrm{eff}}}}}(n_{\mathrm{M}}^2 - 1)\frac{{\left| {\mathop {\smallint }\nolimits_0^{t/2} [(n_{\mathrm{M}}^2 + 1)D_{r,1}^ \ast D_{r,2}{\mathrm{/}}(2n_{\mathrm{M}}^2\varepsilon _0^2) + E_{z,1}^ \ast E_{z,2} - E_{\theta ,1}^ \ast E_{\theta ,2}]_{r = D/2}zdz} \right|}}{{\sqrt {{\int} c (E_{r,1}B_{z,1}^ \ast - E_{z,1}B_{r,1}^ \ast )drdz} \sqrt {{\int} c (E_{r,2}B_{z,2}^ \ast - E_{z,2}B_{r,2}^ \ast )drdz} }}(\frac{\pi }{2} - \alpha )$$

Given a target hybridization wavelength, modes in symmetric resonators with different thicknesses can be simulated to find the degeneracy point, and the mode coupling in the asymmetric case can be estimated from the above overlap integral without actually simulating the asymmetric resonators. For 780-nm-wavelength silica resonators (using *n*_M_ = 1.454), the prefactor in *g*_c_ is 15.78 GHz, or 0.275 GHz per degree angle. This estimate agrees with the full simulation results for wedge resonators for *α* close to 90° (Fig. 4e).

## Supplementary information

Supplementary Information

## Data Availability

The data that support the plots within this paper and the other findings of this study are available from the corresponding author upon reasonable request.

## Code availability

The code that supports the plots within this paper and the other findings of this study is available from the corresponding author upon reasonable request.
