# Supplementary material for: Dirac solitons in optical microresonators
Source: Light Sci Appl. 2020 Dec 23;9:205. doi: 10.1038/s41377-020-00438-w (PMC7758338; doi:10.1038/s41377-020-00438-w)
Supplement: Supplementary file 1 — Supplementary Information [file 41377_2020_438_MOESM1_ESM.pdf]

Dirac Solitons in Optical Microresonators: Supplementary information

Heming Wang[1], Yu-Kun Lu[1], Lue Wu[1], Dong Yoon Oh[1], Boqiang Shen[1], Seung Hoon Lee[1], and Kerry Vahala[1,†]
[1]T. J. Watson Laboratory of Applied Physics, California Institute of Technology, Pasadena, California 91125, USA.
[†]Corresponding author: vahala@caltech.edu

The conservative coupled Lugiato-Lefever equations may admit solutions with nonzero backgrounds, where the fields do not vanish when $\theta \to \pm\infty$. In the following we will show the existence of these solutions with the help of a phase space and then derive some special cases of such solutions. We note that, while these solutions are valid for the conservative hybrid-mode system, the addition of loss or other broadband effects may change the solutions in a qualitative way. The background fields also make the solutions difficult to satisfy the periodic conditions for a resonator. It is not known if soliton solutions with backgrounds can exist in a lossy resonator in the form given below.

The equations for the Dirac soliton reads

$$(\delta D_1 - v)\partial_\theta E_1 = -i\delta\omega E_1 + ig_c E_2 + i(g_{11}|E_1|^2 E_1 + g_{12}|E_2|^2 E_1) \tag{S1}$$

$$-(\delta D_1 + v)\partial_\theta E_2 = -i\delta\omega E_2 + ig_c E_1 + i(g_{22}|E_2|^2 E_2 + g_{12}|E_1|^2 E_2) \tag{S2}$$

As in the main text, we introduce the following quantities:

$$\bar{H} = -\delta\omega(|E_1|^2 + |E_2|^2) + g_c(E_1^* E_2 + E_2^* E_1) + \frac{1}{2}\left(g_{11}|E_1|^4 + g_{22}|E_2|^4 + 2g_{12}|E_1|^2|E_2|^2\right) \tag{S3}$$

$$\bar{N} = (\delta D_1 - v)|E_1|^2 - (\delta D_1 + v)|E_2|^2 \tag{S4}$$

$$G = \frac{\delta D_1 + v}{\delta D_1 - v}\frac{g_{11}}{2} + \frac{\delta D_1 - v}{\delta D_1 + v}\frac{g_{22}}{2} + g_{12} \tag{S5}$$

We begin by obtaining the background (continuous-wave) solutions in the system. To eliminate the global phase dependence, we rewrite the equations of motion using two amplitude variables, $|E_1|$ and $|E_2|$, and a phase difference variable, $\chi \equiv \arg(E_1 E_2^*)$:

$$(\delta D_1 - v)\partial_\theta |E_1| = g_c |E_2| \sin\chi \tag{S6}$$

$$(\delta D_1 + v)\partial_\theta |E_2| = g_c |E_1| \sin\chi \tag{S7}$$

$$\partial_\theta \chi = -\frac{2\delta D_1 \delta\omega}{\delta D_1^2 - v^2} + \left(\frac{g_c}{\delta D_1 - v}\frac{|E_2|}{|E_1|} + \frac{g_c}{\delta D_1 + v}\frac{|E_1|}{|E_2|}\right)\cos\chi + \left(\frac{g_{11}|E_1|^2 + g_{12}|E_2|^2}{\delta D_1 - v} + \frac{g_{22}|E_2|^2 + g_{12}|E_1|^2}{\delta D_1 + v}\right) \tag{S8}$$

We denote the background solutions as $|E_1|_0$, $|E_2|_0$ and χ_0, and at these points all three derivatives should vanish. This happens when $|E_1|_0$ and $|E_2|_0$ are both zero, or are both nonzero. As we have solved the first case in the previous section, we will focus on the case where $|E_1|_0 > 0$ and $|E_2|_0 > 0$. In this case $\sin\chi_0 = 0$, and $\chi_0 = 0$ or π, i.e. the two components in the background are completely in-phase or out-of-phase relative to the mode coupling.

A two-dimensional phase space can be constructed from the real and imaginary parts of $E_1 E_2^*$ (Fig. S1a). The fields at each θ correspond to a point in the diagram, and follow a contour defined by constant $\bar{H}$ and $\bar{N}$ as θ varies. Background solutions appear in the diagram as fixed points on the real axis. Soliton solutions converge to the background for $\theta \to \pm\infty$, and therefore are homoclinic orbits connecting the background state to itself (Fig. S1b). The shape of the orbit is a limaçon and is described by the following equation:

$$\left[zz^* + \frac{a}{2}(z + z^*)\right]^2 = b^2 zz^*, \quad z = E_1 E_2^* - |E_1|_0 |E_2|_0 \cos\chi_0 \tag{S9}$$

$$a = \frac{2g_c}{G}(1 + G|E_1|_0|E_2|_0\cos\chi_0/g_c), \quad b = \frac{g_c}{|G|}\frac{|(\delta D_1 - v)|E_1|_0^2 + (\delta D_1 + v)|E_2|_0^2|}{|E_1|_0|E_2|_0}\sqrt{\frac{1 + G|E_1|_0|E_2|_0\cos\chi_0/g_c}{\delta D_1^2 - v^2}} \tag{S10}$$

According to the properties of a limaçon, when $b < |a|$ the curve has a inner loop, and the background solution becomes a saddle point (Fig. S1b). The inner loop and the outer loop each correspond to a soliton solution, where the

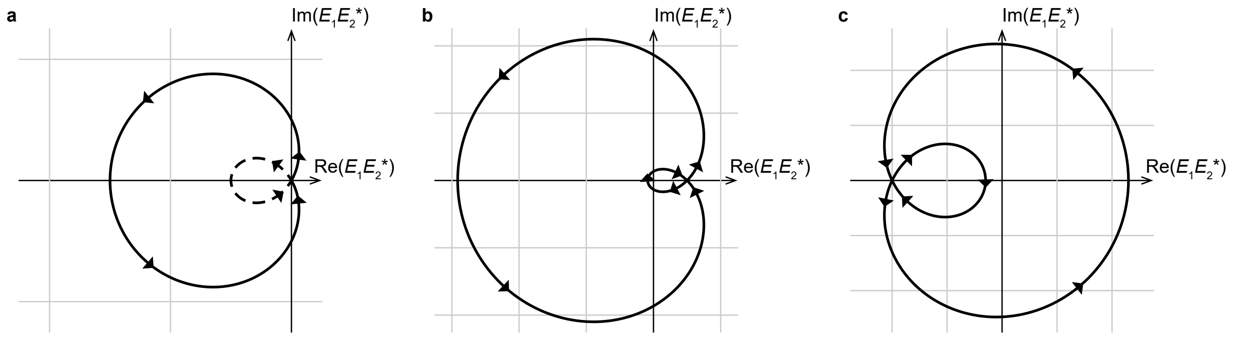

FIG. S1: Phase space portraits of solitons in the hybrid-mode system. For simplicity we choose $g_{11} = g_{22} = 0$ ($G = g_{12}$) in these plots. The length of one grid unit in the plot represents $2g_c/G$. Arrows indicate the direction of state change when θ increases. (a) The phase space portrait for bright solitons with $v = 0$, $\delta\omega = -g_c/2$ (dashed line) and $v = 0$, $\delta\omega = g_c/2$ (solid line). (b) The phase space portrait for dark soliton and soliton-on-background solutions, with a component-in-phase background. Parameters are $v = 0$, $\delta\omega = 2g_c$ and $|E_1|_0^2 = |E_2|_0^2 = g_c/G$. (c) The phase space portrait for dark soliton and soliton-on-background solutions, with a component-out-of-phase background. Parameters are $v = -5/3\delta D_1$, $\delta\omega = 3g_c$ and $|E_1|_0^2 = |E_2|_0^2 = 4g_c/G$. In both (b) and (c) the saddle point topology is present near the background state.

inner loop resembles the conventional dark soliton and the outer loop is a soliton-on-background solution. If $b > |a|$ the limaçon is a simple closed curve that does not pass through the background state, and the solution becomes a Turing roll. For the critical case $b = |a|$, the limaçon reduces to a cardioid, and only the soliton-on-background solution remains.

The sign of $\cos\chi_0$ determines if the background components are in-phase or out-of-phase, and how the limaçon is oriented. For $|v| < \delta D_1$, the $b \le |a|$ condition results in $\chi_0 = 0$. In this case the reduced detuning is restricted to $\tilde{\xi} \ge 1$, and the resonance line of the soliton intersects the bottom branch twice. For $|v| > \delta D_1$, $\cos\chi_0$ has the opposite sign to G, which may become negative. No particular restrictions have been found for the detuning $\delta\omega$, and the resonance line of the soliton intersects both branches once. Typical phase spaces of these two cases are illustrated in Figs. S1b and S1c. The case $|v| = \delta D_1$ does not correspond to solitons, as one of the $|E_{1,2}|$ loses its dynamics, and all solutions are continuous waves.

In the following, we derive the analytical solutions for these solitons. We restrict ourselves to the case $|v| < \delta D_1$ to avoid the discussions on parameters that may change sign, but the technique can be readily generalized. We introduce additional reduced variables to simplify the expressions:

$$\tilde{E_1} \equiv \sqrt{\delta D_1 - v}E_1, \qquad \tilde{E_2} \equiv \sqrt{\delta D_1 + v}E_2, \qquad \tilde{G} \equiv \frac{G}{g_c}|E_1|_0|E_2|_0 \tag{S11}$$

Similarly, $|\tilde{E_1}|_0$ and $|\tilde{E_2}|_0$ are the values of the corresponding variable at the background.

We extend the definition of ψ^2 as

$$\psi^2 \equiv \frac{1}{2}(|\tilde{E_1}|^2 + |\tilde{E_2}|^2) = \frac{1}{2}\left[(\delta D_1 - v)|E_1|^2 + (\delta D_1 + v)|E_2|^2\right] \tag{S12}$$

which has the same meaning as the ψ^2 in the main text when $\bar{N} = 0$. The value of ψ^2 at the background reads $\psi_0^2 \equiv \left[(\delta D_1 - v)|E_1|_0^2 + (\delta D_1 + v)|E_2|_0^2\right]/2$. The differential equation for ψ^2 reads

$$\partial_\theta \psi^2 = 2|E_1||E_2|\sin\chi \tag{S13}$$

$$= \frac{\delta D_1}{\sqrt{\delta D_1^2 - v^2}}(\psi^2 - \psi_0^2)\sqrt{4(1+\tilde{G}) - \frac{[\tilde{G}(\psi^2 - \psi_0^2) - 2\psi_0^2]^2}{|\tilde{E_1}|_0^2|\tilde{E_2}|_0^2}} \tag{S14}$$

where we have used the conservation of $\bar{H}$ and $\bar{N}$ and substituted their values at the background. Integration gives

$$\psi^2 = \psi_0^2 + \frac{2\left[\psi_0^4 - (1+\tilde{G})|\tilde{E_1}|_0^2|\tilde{E_2}|_0^2\right]}{\tilde{G}\left[\psi_0^2 + \sigma\sqrt{1+\tilde{G}}|\tilde{E_1}|_0|\tilde{E_2}|_0\cosh(\beta\tilde{\theta})\right]}, \quad \beta \equiv \sqrt{4\tilde{G} - \frac{\bar{N}^2}{|\tilde{E_1}|_0^2|\tilde{E_2}|_0^2}}, \quad \tilde{\theta} = \frac{g_c}{\sqrt{\delta D_1^2 - v^2}}\theta \tag{S15}$$

The saddle point criterion from the limaçon ensures that β is a real number. The σ before the cosh function is determined by how the square root is taken. For dark-soliton-like solutions (inner loop of the limaçon) we take $\sigma = 1$, and for soliton-on-background solutions (outer loop of the limaçon) we take $\sigma = -1$.

The rest of the solution process is identical to the bright soliton case, which proceeds by finding the equation for $\arg E_{1,2}$ followed by integration. Combining all results above, the field solution can be written as

$$\begin{aligned} E_1 = & \left[|E_1|_0^2 - \frac{|\tilde{E}_1|_0|\tilde{E}_2|_0\beta^2\cosh(\beta\tilde{\theta}) + i(\bar{N} + 2\tilde{G}|\tilde{E}_1|_0^2)\beta\sinh(\beta\tilde{\theta})}{(\delta D_1 - v)\tilde{G}\left[2\sigma\sqrt{1+\tilde{G}} + 2\psi_0^2/(|\tilde{E}_1|_0|\tilde{E}_2|_0)\cosh(\beta\tilde{\theta}) + i\beta\sinh(\beta\tilde{\theta})\right]} \right]^{1/2} \\ & \times \left[\frac{2\sigma\sqrt{1+\tilde{G}} + 2\psi_0^2/(|\tilde{E}_1|_0|\tilde{E}_2|_0)\cosh(\beta\tilde{\theta}) - i\beta\sinh(\beta\tilde{\theta})}{2\sigma\sqrt{1+\tilde{G}}\cosh(\beta\tilde{\theta}) + 2\psi_0^2/(|\tilde{E}_1|_0|\tilde{E}_2|_0)} \right]^{\gamma/2} \exp(ik_0\theta) \end{aligned} \tag{S16}$$

$$\begin{aligned} E_2 = \pm & \left[|E_2|_0^2 - \frac{|\tilde{E}_1|_0|\tilde{E}_2|_0\beta^2\cosh(\beta\tilde{\theta}) + i(\bar{N} + 2\tilde{G}|\tilde{E}_2|_0^2)\beta\sinh(\beta\tilde{\theta})}{(\delta D_1 + v)\tilde{G}\left[2\sigma\sqrt{1+\tilde{G}} + 2\psi_0^2/(|\tilde{E}_1|_0|\tilde{E}_2|_0)\cosh(\beta\tilde{\theta}) + i\beta\sinh(\beta\tilde{\theta})\right]} \right]^{1/2} \\ & \times \left[\frac{2\sigma\sqrt{1+\tilde{G}} + 2\psi_0^2/(|\tilde{E}_1|_0|\tilde{E}_2|_0)\cosh(\beta\tilde{\theta}) - i\beta\sinh(\beta\tilde{\theta})}{2\sigma\sqrt{1+\tilde{G}}\cosh(\beta\tilde{\theta}) + 2\psi_0^2/(|\tilde{E}_1|_0|\tilde{E}_2|_0)} \right]^{\gamma/2} \exp(ik_0\theta) \end{aligned} \tag{S17}$$

$$k_0 \equiv \frac{1}{2\delta D_1}\left(g_c \frac{|E_2|_0^2 - |E_1|_0^2}{|E_1|_0|E_2|_0} + (g_{11} - g_{12})|E_1|_0^2 - (g_{22} - g_{12})|E_2|_0^2 \right) \tag{S18}$$

where the sign of E_2 is negative if the limaçon loop encloses the origin, or positive if the origin is not enclosed. $|E_1|_0$ and $|E_2|_0$ are the background field amplitudes, i.e. the positive solutions to the following equation:

$$2\delta D_1\delta\omega = g_c(\delta D_1 + v)\frac{|E_2|_0}{|E_1|_0} + g_c(\delta D_1 - v)\frac{|E_1|_0}{|E_2|_0} + (g_{11}|E_1|_0^2 + g_{12}|E_2|_0^2)(\delta D_1 + v) + (g_{22}|E_2|_0^2 + g_{12}|E_1|_0^2)(\delta D_1 - v) \tag{S19}$$

A special case can be obtained by setting $g_{11} = g_{22}$, $v = 0$, and $|E_1|_0 = |E_2|_0 = \sqrt{(\delta\omega - g_c)/(g_{11} + g_{12})}$. In this case

$$E_1 = -E_2^* = \sqrt{\frac{\delta\omega - g_c}{g_{11} + g_{12}}}\,\frac{\sqrt{\delta\omega - g_c} - i\sigma\sqrt{\delta\omega}\sinh(2\sqrt{(\delta\omega - g_c)g_c}\theta/\delta D_1)}{\sqrt{\delta\omega}\cosh(2\sqrt{(\delta\omega - g_c)g_c}\theta/\delta D_1) + \sigma} \tag{S20}$$
